# Direct oral anticoagulants: the safer choice in chronic kidney disease?

**DOI:** 10.1093/ckj/sfad288

**Published:** 2023-11-20

**Authors:** Thomas A Mavrakanas

**Affiliations:** Division of Nephrology, Department of Medicine, McGill University Health Centre, Montreal, Quebec, Canada; MUHC Research Institute, Montreal, Quebec, Canada | 2B.44–5252 de Maisonneuve W, Montreal, QC, Canada

Atrial fibrillation is the most common arrhythmia and represents a major cause of ischemic stroke. Anticoagulation for stroke prevention is the mainstay of medical management for patients with this condition. Vitamin K antagonists (VKAs) were introduced in medical practice in the 1950s and were until recently the most widely used anticoagulants for this indication. However, use of VKAs requires routine coagulation monitoring and frequent dose adjustment. In addition, these agents have a narrow therapeutic index and numerous food and drug interactions.

To overcome these limitations, direct oral anticoagulants (DOACs) were developed. They target factor II (dabigatran) or factor X (apixaban, edoxaban, rivaroxaban), have a rapid onset of action, minimal interactions with food or other drugs, and do not require routine coagulation monitoring and dose adjustment [1]. In large randomized clinical trials, all four DOACs were noninferior or superior to VKAs for the efficacy outcome of stroke and systemic embolism or for the safety outcome of major bleeding. Since then, these agents have largely replaced VKAs for most patients with atrial fibrillation.

Another important consideration is change in renal function over time while on oral anticoagulation. This issue, although somewhat inconspicuous, gained significant interest after the description of anticoagulant-related nephropathy. This entity was initially reported in patients presenting with hematuria and unexplained acute kidney injury (AKI) who were treated with warfarin and had supratherapeutic anticoagulation [2]. It was subsequently encountered in patients treated with DOACs [3]. The pathophysiology involves obliteration of the Bowman space and renal tubules by red cell casts. Findings from biopsy series have been reproduced in animal models of warfarin-related nephropathy [4]. Other pathogenic mechanisms explaining a potential link between the choice of anticoagulant agent and kidney outcomes include hemodynamic renal injury from overt bleeding or accelerated vascular calcification with VKAs.

**Figure 1:**
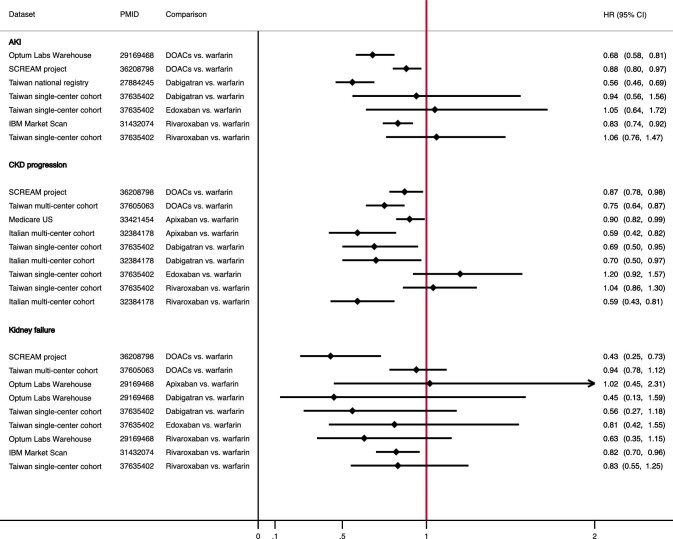
Kidney outcomes in observational studies with direct oral anticoagulants.

DOACs offer a more predictable anticoagulant response and it was hypothesized that the incidence of AKI would be lower with these agents, compared with VKAs. It was also hypothesized that DOAC use might result in slower glomerular filtration rate (GFR) decline over time because AKI is associated with development and progression of chronic kidney disease (CKD). This hypothesis was tested in three large randomized clinical trials with DOACs in the general population (Randomized Evaluation of Long-Term Anticoagulation Therapy-RE-LY, Rivaroxaban Once Daily Oral Direct Factor Xa Inhibition Compared with Vitamin K Antagonism for Prevention of Stroke and Embolism Trial in Atrial Fibrillation-ROCKET-AF, and Apixaban for Reduction in Stroke and Other Thromboembolic Events in Atrial Fibrillation-ARISTOTLE). A slower rate of GFR decline was observed with dabigatran but not with rivaroxaban or apixaban [5–7]. However, these analyses were *post hoc* in nature and validation of these findings in large patient cohorts was deemed necessary. Findings from observational studies on the potential association between the choice of oral anticoagulant and the incidence of AKI and CKD progression or kidney failure are shown in Fig. 1. In summary, most of the studies demonstrated a protective effect from DOACs compared with VKAs on all endpoints. Nevertheless, the effect size was implausibly large in many of these studies, while outcomes were identified using administrative diagnostic codes or laboratory data that were available only during hospitalization. In addition, the follow-up period was relatively short, limiting the confidence on findings with respect to CKD progression. Therefore, whether the choice of oral anticoagulant has any effect on renal outcomes remains uncertain.

In this issue of the *Clinical Kidney Journal*, Vestergaard *et al*. report their findings on the risk of AKI and CKD in patients with atrial fibrillation initiating a DOAC or a VKA [8]. To study this association, the authors used nationwide data from the Danish National Prescription Registry. They assembled a retrospective cohort of 32 781 new users of oral anticoagulants between 2012 and 2018 with a diagnosis of non-valvular atrial fibrillation within 90 days prior to the index date, at least one outpatient creatinine value in the 365 days prior to the anticoagulant prescription and an estimated GFR ≥15 mL/min/1.73 m^2^. Median age at treatment initiation was 75 years and 49% of patients were female. The prevalence of CKD at baseline, defined as an estimated GFR between 15 and 59 mL/min/1.73 m^2^, was 25% but only 735 individuals had an estimated GFR <30 mL/min/1.73 m^2^. A total of 23% was started on a VKA and 77% on a DOAC (41% on apixaban, 37% on rivaroxaban, 20% on dabigatran and 1% on edoxaban). Twenty-six percent of the cohort was also treated with aspirin and 9% with clopidogrel. DOAC use increased from 47% in 2012 to 97% in 2018.

The authors calculated a propensity score for receiving a DOAC vs a VKA and applied inverse probability of treatment weighing (IPTW) to account for differences between the two groups at baseline. The IPTW cohort was well balanced for all baseline characteristics. In the intention-to-treat analysis, the weighted risk of AKI at 1 year was lower in patients treated with DOACs compared with VKAs: hazard ratio (HR) of 0.86 [95% confidence interval (CI) 0.82–0.91], corresponding to 2.1 fewer events per 100 patients. Similarly, the weighted risk of CKD progression at 5 years was lower in patients treated with DOACs: HR of 0.85 (95% CI 0.79–0.92), corresponding to 2.3 fewer events per 100 patients at 5 years. The benefit was even more pronounced for the incidence of kidney failure. In addition, prescription of a DOAC was associated with a lower risk of death from any cause or major bleeding but a similar risk of stroke or embolism, compared with VKAs. Among the three DOACs, dabigatran and apixaban, but not rivaroxaban, were associated with a significantly lower risk of kidney outcomes. Point estimates in the per protocol analysis were similar in magnitude and direction but the CIs were wider and the comparison between the two treatment strategies did not reach statistical significance for any of the studied outcomes. In addition, the treatment effect was similar across different patient subgroups, including those with CKD at baseline.

This is the largest observational study on the risk of kidney disease with DOACs and VKAs in patients with atrial fibrillation. Use of a nationwide cohort with universal healthcare access, adjudication of clinical endpoints on the basis of longitudinal laboratory data and a rigorous methodological approach constitute unique strengths of this study. However, as the authors acknowledge, unmeasured residual confounding may account for at least part of the differences in clinical outcomes between the two groups. Furthermore, 34% of the new users did not have a creatinine value available in the year prior to the DOAC prescription and in 51% of the individuals the indication for oral anticoagulation was unknown. These patients who were not included in the analysis seem to belong to a healthier population, limiting the generalizability of study findings. In addition, the number of individuals with stage 4 CKD was very small and no definite conclusion can be drawn for these patients. Finally, definition of study endpoints merits discussion. The clinical significance of stage 1 AKI is questionable and a change in creatinine levels of only 26.5 μmol/L might simply represent fluctuation around baseline, especially in patients with underlying CKD. In addition, decline in estimated GFR may occasionally be due to the introduction of nephroprotective agents, such as renin–angiotensin–aldosterone system blockers or sodium–glucose co-transporter 2 inhibitors. However, a similar trend for the hard endpoint of kidney failure is suggestive of slower GFR decline among DOAC users.

Several additional points need to be raised. First, efficacy and safety of VKAs depends on time in therapeutic range, which is harder to achieve as kidney function declines. Second, appropriate dose adjustment is necessary for all DOACs in patients with CKD. However, the optimal dose for each DOAC in advanced CKD has not been accurately defined [9]. More data are required in this subgroup that was under-represented in the study. Third, the exact mechanism underlying a potential benefit, if any, from DOACs on kidney outcomes is unclear. In addition to a lower incidence of hemodynamic injury to the kidney in the setting of a major bleeding event or possibly a lower incidence of anticoagulant-related nephropathy, whose exact clinical significance is still unknown, the observed benefit could also be due simply to higher use of nephroprotective agents among patients on DOACs, reflecting differential prescription patterns between physicians who use DOACs or VKAs rather than a direct treatment effect. Fourth, regardless of a potential effect on the kidney, there is established clinical benefit from all four DOACs which were proven to be noninferior or superior to VKAs with respect to stroke prevention or major bleeding in large randomized trials in the general population. These trials also enrolled more than 12 000 patients with stage 3 CKD and showed similar results in this subgroup. Finally, in line with prescription patterns identified elsewhere, in this Danish cohort use of DOACs appropriately increased from 47% to 97% of eligible patients between 2012 and 2018. Therefore, the question of potential kidney benefits with DOACs is mostly of historical significance and the practice has already shifted.
